# Chemical Elemental Distribution and Soil DNA Fingerprints Provide the Critical Evidence in Murder Case Investigation

**DOI:** 10.1371/journal.pone.0020222

**Published:** 2011-06-03

**Authors:** Giuseppe Concheri, Daniela Bertoldi, Elisa Polone, Stefan Otto, Roberto Larcher, Andrea Squartini

**Affiliations:** 1 Dipartimento di Biotecnologie Agrarie, Università di Padova, Legnaro (Padova) Italy; 2 National Research Council (CNR), Institute of Agro-Environmental and Forest Biology, Legnaro (Padova) Italy; 3 IASMA – Edmund Mach Foundation, San Michele all'Adige (Trento) Italy; New England Biolabs, Inc., United States of America

## Abstract

**Background:**

The scientific contribution to the solution of crime cases, or throughout the consequent forensic trials, is a crucial aspect of the justice system. The possibility to extract meaningful information from trace amounts of samples, and to match and validate evidences with robust and unambiguous statistical tests, are the key points of such process. The present report is the authorized disclosure of an investigation, carried out by Attorney General appointment, on a murder case in northern Italy, which yielded the critical supporting evidence for the judicial trial.

**Methodology/Principal Findings:**

The proportional distribution of 54 chemical elements and the bacterial community DNA fingerprints were used as signature markers to prove the similarity of two soil samples. The first soil was collected on the crime scene, along a corn field, while the second was found in trace amounts on the carpet of a car impounded from the main suspect in a distant location. The matching similarity of the two soils was proven by crossing the results of two independent techniques: a) elemental analysis via inductively coupled plasma mass spectrometry (ICP-MS) and optical emission spectrometry (ICP-OES) approaches, and b) amplified ribosomal DNA restriction analysis by gel electrophoresis (ARDRA).

**Conclusions:**

Besides introducing the novel application of these methods to forensic disciplines, the highly accurate level of resolution observed, opens new possibilities also in the fields of soil typing and tracking, historical analyses, geochemical surveys and global land mapping.

## Introduction

Although several names have been coined referring to the use of geochemical data to assist forensic investigation and judgement [1], [2], this discipline is still in a rather unofficial stage and the potentialities of connection between soil science and law remain largely unexploited [3].

Nevertheless soil and mud particles carried over passively from outdoor sites are often associated to human walking outfit, as well as car tyres, fenders and mats. Such particles contain a wealth of information about the places visited by the people carrying or loosing them.

Although soil granules abound on crime scenes worldwide, they are seldom envisaged as a possible exhibit in court trials and their potential role of evidence in forensic context is largely overlooked, often due to their very low amount [4]. On the contrary, given the soil composition complexity in chemical and biological terms, the inherent degree of information could warrant the achievement of a highly site-specific fingerprint. In the present communication we report the application of two soil characterization techniques in a murder case occurred in Italy, on which we worked by appointment of the Attorney General.

## Methods

### Mineral element analyses

#### Chemicals

MilliQ water (Millipore, Bedford, MA, USA), nitric acid at 69.5% (Superpure; Merck, Darmastadt, Germany), hydrochloric acid at 37% (ACS; Riedel-deHaën, Seelze, Germany) and hydrogen peroxide at 30% (Superpure; Merck, Darmstadt, Germany) were used. ICP Multielement Standard Solution VI (Merck), Multielement Calibration Standard 1 (Agilent Technologies, Santa Clara, CA, USA), Multielement Calibration Standard 3 (Agilent Technologies, Santa Clara, CA, USA), ICP Multielement Standard Solution 4 (Aristar, BDH, Poole, UK) and mono-element standard solution for Cs (1 g/ml; Ultra Scientific, Bologna, Italy), Fe (10 mg/ml; CPI international, Santa Rosa, CA, USA) P, S, Cu and Mn (all 1 g/ml; Merck, Darmstadt, Germany) were used to prepare external standard solutions whereas 4 mono-element solution of Rh, Sc, Tb and Re (1 mg/ml, Aristar, BDH, Poole, UK) were used to prepare internal standard solutions. All standard solutions were diluted and stabilized with the addition of a 1% HNO_3_ and 0.2% HCl solution. The accuracy was proven using as reference material a soil provided by the “Wageningen evaluating programs for analytical laboratories” All the materials used were previously washed with nitric acid (5%) and rinsed twice with milliQ water.

#### Sample preparation

Soil samples were air-dried, and the <2 mm fraction was ground in order to pass it through a 0.2 mm sieve. Samples (0.25 g) were acid digested in a microwave system (PTFE vessel, MARS EXPRESS, CEM, USA; max temperature 175°C) after the addition of 1.5 ml of H_2_O_2_, 4.5 ml of HCl (37%), 1.5 ml of HNO_3_ (96%) and 0.25 ml of internal standard solution (Re, 80 mg/L).

The digested samples were diluted 40 times before the ICP-MS analysis and 2 times for the ICP-OES analysis.

#### Analysis

53 mineral elements (^109^Ag, ^27^Al, ^75^As, ^11^B, ^137^Ba, ^9^Be, ^209^Bi, ^111^Cd, ^140^Ce, ^40^Ca, ^59^Co, ^52^Cr, ^133^Cs,^ 63^Cu ^163^Dy, ^166^Er, ^151^Eu, ^56^Fe, ^71^Ga, ^157^Gd, ^74^Ge, ^178^Hf, ^202^Hg, ^165^Ho, ^39^K, ^139^La, ^7^Li, ^26^Mg, ^55^Mn, ^98^Mo, ^23^Na, ^146^Nd, ^60^Ni, ^31^P, ^206+207+208^Pb, ^108^Pd, ^141^Pr, ^85^Rb, ^121^Sb, ^78^Se, ^147^Sm, ^118^Sn, ^88^Sr, ^126^Te, ^232^Th, ^49^Ti, ^205^Tl, ^169^Tm, ^238^U, ^51^V, ^89^Y, ^171^Yb, ^66^Zn) were analysed using an ICP-MS (7500ce, Agilent Technologies, Tokyo, Japan) equipped with an autosampler ASX-520 (Cetac Technologies Inc., Omaha, NE, USA). After preparation, the samples were automatically introduced into a Scott spray chamber using a MicroMist nebulizer and then into a Fassel type torch. An Octopole Reaction System (ORS) using He (for As, Cr, Cu, Eu, Fe, K, Mg, Na, Ni, V and Zn quantification) and H_2_ (for Ca, Ga and Se) as collision and reaction gases respectively, was used to remove polyatomic interferences. An on-line solution of Sc, Rh and Tb (3 mg/L) was used as the added on-line internal standard.

Sulphur (S, 181 nm) was quantified using a ICP-OES (Optimal 3300 Dual view, Perkin Elmer; axial-mode) equipped with a cyclonic nebulizer.

### Soil processing for microbiological analyses

Soil from the car carpets was scooped with a spatula into plastic falcon tubes. Soil aliquots of 50–60 g from the crime scene were collected from the top layer in three replicates, stored in plastic bags and transferred to the laboratory in a refrigerated box. Soil samples were air-dried for 3 days at room temperature; soil crumbs were minced with a pestle and the material was sieved through a 1 mm wire mesh grid; replicate aliquots of 300 mg were sampled from each thesis. Total DNA was extracted by using an Ultraclean^TM^ Soil DNA Kit (MoBio, Laboratories, Inc., Solana Beach, CA, USA) following the manufacturer's instructions, with the exception of the shaking incubation time that was prolonged to 60 min.

### Amplified Ribosomal DNA Restriction Analysis (ARDRA) of the bacterial community

One µl of the lysate containing the microbial community DNA extracted as described above, or its serial 1/50 or 1/100 dilution, was treated in a PCR BioRad 170–8740 I-Cycler using the two 16S rDNA-targeted universal bacterial primers 63F 5′CAGGCCTAACACATGCAAGTC) [5] and 1389R (5′ACGGGCGGTGTGTACAAG) [6] at 1 µm each in a 25 µl reaction volume, using the following program: initial denaturation at 94°C for 2 min; 35 cycles at 94°C for 60 sec, 54°C for 30 sec, 72°C for 150 sec and a final extension at 72°C for 5 min. The PCR reaction mixture contained 20 mm Tris-HCl (pH 8.4), 50 mm KCl, 1.5 mm MgCl_2_, 0.2 mm of each dATP, dCTP, dGTP and dTTP, 250 nm of each primer and 1 U Taq DNA Polymerase, recombinant (InVitrogen Life Technologies). Aliquots (5 µl) of the resulting amplicons were visualized by electrophoresis in 1.2% agarose gels stained with ethidium bromide (0.3 µg/ml). 10–15 µl aliquots were digested overnight at 37°C with 15U of *Hin*6I, or with 10U of *Hin*fI, or with *Hap*II enzymes (Amersham Biosciences). Digested DNA was loaded on a 2% agarose gel, run electrophoretically for 3 h at 100V. The ethidium bromide-stained gel was visualized over a UV transilluminator and photographed by a Kodak DC290 digital camera (Kodak, Rochester, NY). Clustering of the three combined electrophoretograms was performed by means of GelComparII software (Applied Maths inc. Kortrijk, Belgium).

### Statistical Analysis for elemental content data

The analytical dataset was a matrix of rows of 29 soil samples by columns of the 54 mineral elements. Data were expressed as soil concentrations, in mg/kg. In order to remove interference due to different ranges of concentration, and facilitate comparison across elements, all analyses were performed on values normalized by their mean and standard deviation. Simple correlations between elements were tested with Pearson's *r* (p = 0.05).

An initial study of variability and possible grouping was performed with simple box and whiskers graph (mean, stand. deviation, stand. error) for each mineral element and for a synthetic variable as a sum of all normalized concentrations to evaluate in a glance the grouping tendency across all elements.

The grouping tendency was recalculated and verified with cluster analysis and other two multivariate exploratory techniques, i.e. Principal components and Discriminant analysis. All statistical analyses were performed with Statistica 7.0 (Statsoft Inc., 2005).

## Results and Discussion

With the task of assessing whether a person suspected of involvement could have been on the crime site, available particles of soil were collected from the carpets of a car impounded from the suspect (3.75 g from the left carpet (CarL) - and 1.98 g from the right one (CarR), and compared with walkable top soil (0–2 cm depth) collected from a path along a corn field in the crime scene.

**Figure 1:**
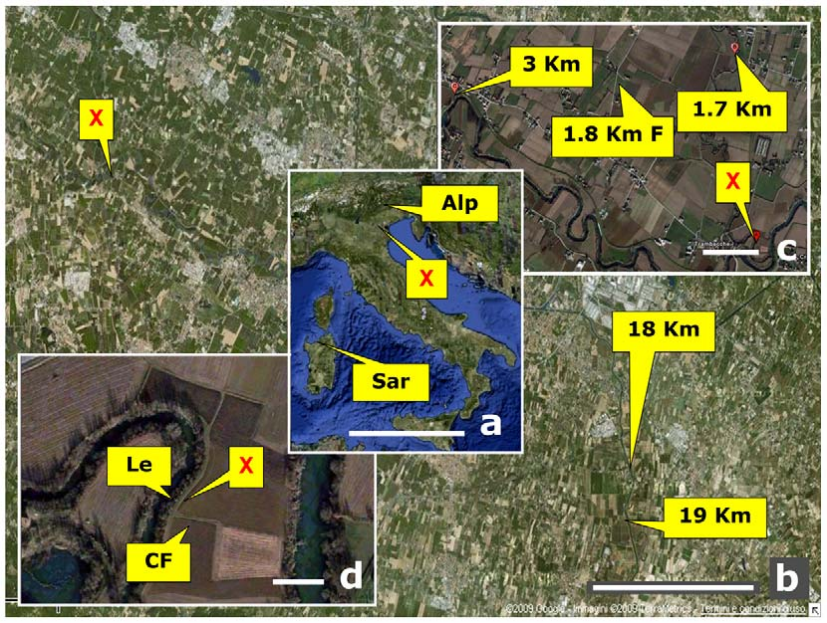
Air view maps of the crime scene and surroundings depicting the sites from which samples were taken, starting from the murder spot, and at increasing distances. The inset map of Italy shows the area location as well as the sites of two soils taken at far distance as outgroup references. X: spot where the corpse was found (margin of a corn field), CF (inside corn field), Le (ridge of the levee bordering the corn field). 1.7 Km, 3 Km, 18 Km, 19 Km: sites located at 1.7, 3, 18, 19 kilometers from the murder site and sharing the same crop (corn); 1.8 KmF: (fallow), site located at 1.8 km but not cropped for over 50 years and featuring natural vegetation and secondary growth. Sar: soil from an uncultivated area in Sardinia (Castelsardo); Alp: soil from an uncultivated area in the Alps (Soranzen). All samples, except the two outgroup references, were chosen in equivalent soil conditions as regards parent material, depositional basin river and soil type (Hypercalcaric-Fluvic Cambisols, WRB 1998, or Oxyaquic Eutrudept fine-silty, carbonatic, mesic, USDA 1998) to minimize the variability that would occur across different soil types. For details and exact coordinates, see Tab.1. Scale bars equal: 500 km (a); 5 km (b); 500 m (c); 50 m (d).

In order to verify the reliability of such comparison, we gathered a series of control soils at increasing distances from the crime scene (Fig. 1). In the near-range, samples were collected from two areas within 30 meters (inside the corn field and along a levee of an adjacent river). Exploring farther locations we sampled from corn field sites at 1.7, 3, 18 and 19 kilometers and a soil with natural vegetation at 1.8 km. Two soils taken at far distances were included as outgroup references: one from the Alps and one from a ruderal coastal area of the Sardinia island. Soil sample collection coordinates are shown in Table 1.

**Table 1:** Location of sampling sites: latitude and longitude coordinates in decimal values.

Site	Description	Latitude	Longitude
**X**	Spot were the corpse was found (margin of a corn field)	N 45.417371°	E 11.728335°
**CF**	Inside corn field	N 40.417187°	E 11.728294°
**Le**	Ridge of the levee bordering the corn field	N 45.417360°	E 11.728178°
**1.7 km**	Site located at 1.7 km from the murder site and sharing the same crop (corn)	N 45.432579°	E 11.725670°
**3 km**	As above at 3 km	N 45.429420°	E 11.693049°
**18 km**	As above 18 km	N 45.348369°	E 11.937301°
**19 km**	As above at 19 km	N 45.328721°	E 11.938243°
**1.8 km F**	Site located al 1.8 km but not cropped for over 50 years and featuring natural vegetation and secondary growth (fallow)	N 45.430336°	E 11.712277°
**Sar**	Control site from an uncultivated area in Sardinia (Castelsardo SS, Italy)	N 40.909556°	E 8.694076°
**Alp**	Control site from an uncultivated area in the Alps (Soranzen, Cesiomaggiore, BL, Italy)	N 46.074211°	E 11.948337°

The “Car” samples were found within the car impounded from the suspect.

Two techniques were applied, the first involving a physical-chemical approach using Inductively Coupled Plasma mass and optical spectrometry (ICP-MS, ICP-OES) to determine the concentration of 54 mineral elements. The second method evaluates the biological variable of soil microbial diversity, assessed by the restriction digestion DNA polymorphism on amplified bacterial ribosomal genes (ARDRA) [7], hereby applied to the whole community [8].

The result from the ICP analysis of the 54 elements was processed for correlation analysis, and 71% of the combinations were significant. The number of pairwise element-element correlation ranged from 50 for Mn, (i.e. Mn content was significantly correlated with that of 50 elements over 53, i.e. not correlated with Mg, V, U) to 6 for Pd (its content correlated only with that oh Hf, Mn, Na, S, Ti, V), with a mean of 38, showing that the dataset contained sufficient and redundant information for a robust analysis run according to statistically stringent procedures [9].

**Figure 2:**
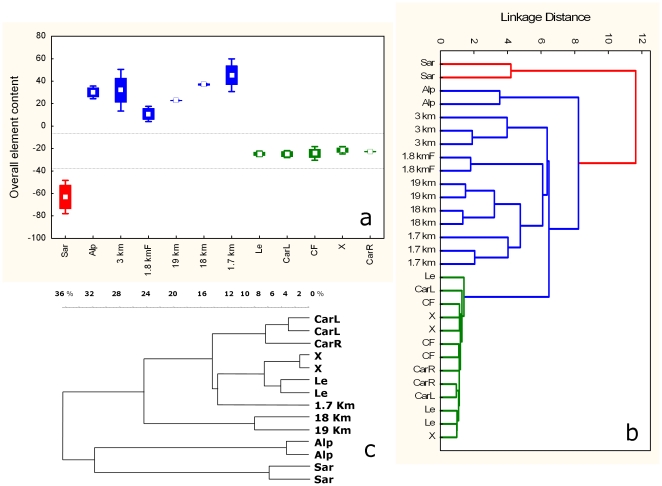
Results of the analyses on the soil samples compared with two specimens of soil (CarR, CarL), found respectively on the right and left carpets of the suspect's car floor. a, b) ICP analysis of the content of 54 mineral elements. a): Box and whiskers synthetic representation of the variability of the 12 zones; b) Cluster analysis (single linkage, Euclidean distance) of the data; c) Amplified Ribosomal DNA Restriction Analysis (ARDRA) of the soil bacterial communities. The Neighbour Joining dendrogram resulting from Pearson correlation analysis of the combined three enzymes electrophoretic profiles is shown. The horizontal scale indicates the percent distance. In b) and c) sample replicates are included to show the degree of inter-replicate variability.

**Figure 3:**
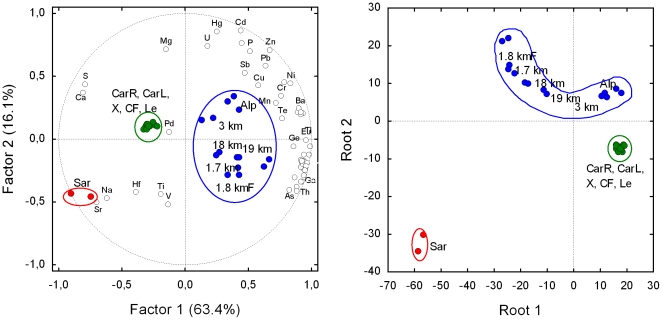
Left: bi-plot after Principal components and classification analysis based on correlation of the content of the 54 chemical elements (empty marker, not all labelled) in the 29 soil samples (full marker). The variance explained by the each principal component is shown in parentheses. Right: plot of canonical scores after Discriminant analysis with canonical analysis performed with the four chemical elements most, and significantly, correlated (absolute value) with factor 1 (Cs, Ga, Ca, S) and factor 2 (Cd, Zn, Hg, Sr). Classification of soil samples has an high probability to be correct (93%) as the predicted classification deviates only for sample “Le”, that is not distinct from “X” (see fig. 1d). Since these two samples are in the same group (the green one), group classification is 100% correct. For technical details of multivariate techniques see Statistica 7.0 Electronic Manual (Statsoft Inc., 2005).

The overall element normalized content in the soils is shown in the box and whiskers graph in Fig. 2a. Samples fell in three non-overlapping groups: the Sardinian soil, the three soils from the crime scene together with the two found on the car carpets, and a third group with the soils from the farther sites. Among the individual element distribution analyses (not shown) some resulted particularly informative, i.e. lithium gave a degree of resolution almost as high as that obtained with the whole set of elements. Results of multivariate exploratory techniques allowed again the grouping of the samples, confirming that the soil found in the car matched with those from the crime scene, and that soils collected within a near range shared a correspondingly high similarity. Fig. 2b shows the Cluster analysis output. The same grouping was obtained with Principal component and classification analysis and Discriminant analysis with canonical analysis (Fig.3).

DNA fingerprinting methods of soil microbial communities can provide an independent approach based on local vs. global biodiversity. The efficiency of PCR amplification of given genes from DNA extracted from soil depends on different critical factors including microbial abundance and the presence of humic or plant compounds that can inhibit DNA polymerase in vitro activity. Nevertheless we were able to amplify bacterial DNA (16S ribosomal gene) from the majority of the samples under study and to compare restriction digestion fingerprints. The tree diagram obtained (Fig. 2c) agrees already by visual assessment with that from the independent approach based on elemental concentration, supporting the highest similarity of samples from the same site and the progressive divergence of the other soils. The ARDRA technique qualifies in this respect as complementary to the elemental analysis. Soil type has been recognized as the primary determinant of microbial community compositions [10]. In addition the profile of bacterial communities is known to be affected by soil pH [11], [12] as well as slope factor [8], and changes along environmental gradients [13].

As regards the strengths of the bacterial community DNA fingerprint technique we refer in particular to [8], in which we could prove the reliability of the method on alpine spruce forest stands in which the different bacterial assemblages could be clearly differentiated according to parameters as parent bedrock (acid vs. basic) , slope (northern vs southern exposure) and stand age of the woods, in this hierarchical order.

In general an analysis of living communities can be considered more sensitive to changes of vegetation and timewise recent effects, while the chemical elemental content reflects primarily each site's long term geology.

Results indicate that the approaches and the results representation used are reliable tools in assisting forensic science and can highly increase its power of resolution with respect to its current levels of knowledge [14], [15]. Specifically, the results of this analysis provided a key evidence for the case conclusion in the judicial Court. While care must be always exerted in considering that the results of these studies are time- and space-scale dependent, the method qualifies as not only suitable to service the needs of geoforensics but also fit for many other applications aiming at soil characterization. An unexpected high level of consistency in the differences among soils appears indeed to exist and suggests many perspective uses of these methods also in soil typing, soil tracking, geochemical studies, historical analyses, and earth-wide mapping projects.
